# Extrapleural Hematoma: A Rare Sequalae of Thoracic Trauma

**DOI:** 10.7759/cureus.70506

**Published:** 2024-09-30

**Authors:** Takshak Shankar, Salva Ameena MS, Vempalli Nagasubramanyam, Rinku Meena, Parvathy Sasidharan

**Affiliations:** 1 Emergency Medicine, All India Institute of Medical Sciences, Rishikesh, IND; 2 Emergency Medicine, All India Institute of Medical Sciences, New Delhi, IND; 3 Trauma Surgery, All India Institute of Medical Sciences, Gorakhpur, IND

**Keywords:** blunt chest trauma, chest trauma, extrapleural hematoma, intercostal drainage, video-assisted thoracic surgery (vats)

## Abstract

Extrapleural hematoma is a rare consequence of thoracic trauma, which is the result of bleeding between the parietal pleura and the endothoracic fascia and is usually diagnosed within the initial 24-48 hours of the injury. Delayed presentations are rarely seen. An elderly male, who was not on any antiplatelet or anticoagulant medications, presented to the emergency department six days after sustaining a trivial blunt chest trauma with a large right extrapleural hematoma. He was successfully treated with video-assisted thoracoscopic surgery. Geriatric patients must be followed up with serial chest radiographs to monitor the development of these rare complications.

## Introduction

A rare consequence of thoracic trauma is an extrapleural hematoma, with a reported incidence of 7.1%. Most of these cases result from blunt trauma to the chest and are found in association with rib fractures, hemothoraces, pneumothoraces, and pulmonary and chest wall contusions [1]. An extrapleural hematoma occurs due to bleeding between the parietal pleura and the endothoracic fascia [2]. The diagnosis of an extrapleural hematoma is usually made radiologically within the initial 24-48 hours of the injury [1,3]. A delayed presentation with physiological alterations is rare and often requires surgical intervention for treatment [1]. We present a unique case of an elderly male who sustained minor blunt chest trauma and presented to our hospital six days later with a large right extrapleural hematoma.

## Case presentation

An elderly male in his late seventies, an avid smoker with a history of hypertension and no other comorbidities, presented to the emergency department (ED) with complaints of right-sided chest pain and shortness of breath. Six days prior, he had slipped and fallen from his bed while disembarking, striking his chest, and experiencing moderate to severe pain since then. He was physically active at baseline and was not taking any antiplatelet or anticoagulant medications. The chest pain had been managed with over-the-counter analgesics, including ibuprofen and paracetamol. The shortness of breath developed one day ago; he was dyspneic at rest but remained conscious and alert.

Upon arrival in the ED, his vital signs were as follows: a pulse rate of 64 beats per minute, blood pressure of 100/70 mm Hg, and peripheral oxygen saturation of 80% on room air, which improved to 94% with oxygen administered at four liters per minute. His respiratory rate was 24 breaths per minute. Physical examination revealed a positive chest compression test on the right side, significantly reduced air entry, and dullness to percussion. Other systemic examinations were within normal limits.

**Figure 1:**
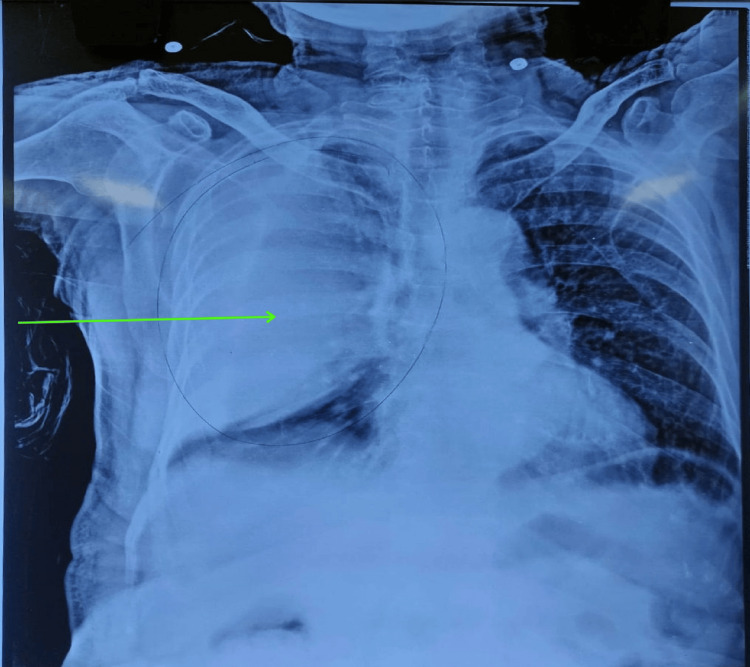
Chest X-ray of the patient showing a D-shaped opacity on the right side with its base toward the chest wall and sparing of the costophrenic angle (green arrow)

An extended physical examination suggested a massive effusion on the right side, likely a hemothorax. The patient was resuscitated with intravenous fluids, and blood products were arranged. Injection tramadol was administered for pain relief. He was planned for intercostal tube drainage. Meanwhile, a bedside chest X-ray was performed as per protocol, which revealed a D-shaped opacity on the right side (Figure 1).

Basic laboratory investigations were unremarkable (Table 1).

**Table 1:** Laboratory investigations of the patient on arrival HB: hemoglobin, CBC: complete blood count, TLC: total leukocyte count, DLC: differential leukocyte count, SGOT: serum glutamate oxaloacetate transaminase, SGPT: serum glutamate pyruvate transaminase, aPTT: activated partial thromboplastin time, PT INR: prothrombin time international normalized ratio

Laboratory parameters	Laboratory investigations on arrival	Reference range
HB (g/dL)	9.6	11.5-15
TLC (cells/mm3)	14900	4000-10000
DLC (%)	N78, L32	-
Platelet count (thousand/mm3)	154	150-450
Serum potassium (mmol/L)	3.98	3.5-5.1
Serum sodium (mmol/L)	136	136-146
Urea (mg/dL)	24.7	10-43
Creatinine (mg/dL)	0.9	0.5-1
Total bilirubin (mg/dL)	0.83	0.3-1.2
Direct bilirubin (mg/dL)	0.11	<0.2
SGOT (U/L)	36	<35
SGPT (U/L)	32	<35
Serum albumin (g/dL)	3.61	3.5-5.2
aPTT (seconds)	29	25-35
PT INR	0.98	0.8-1.1

**Figure 2:**
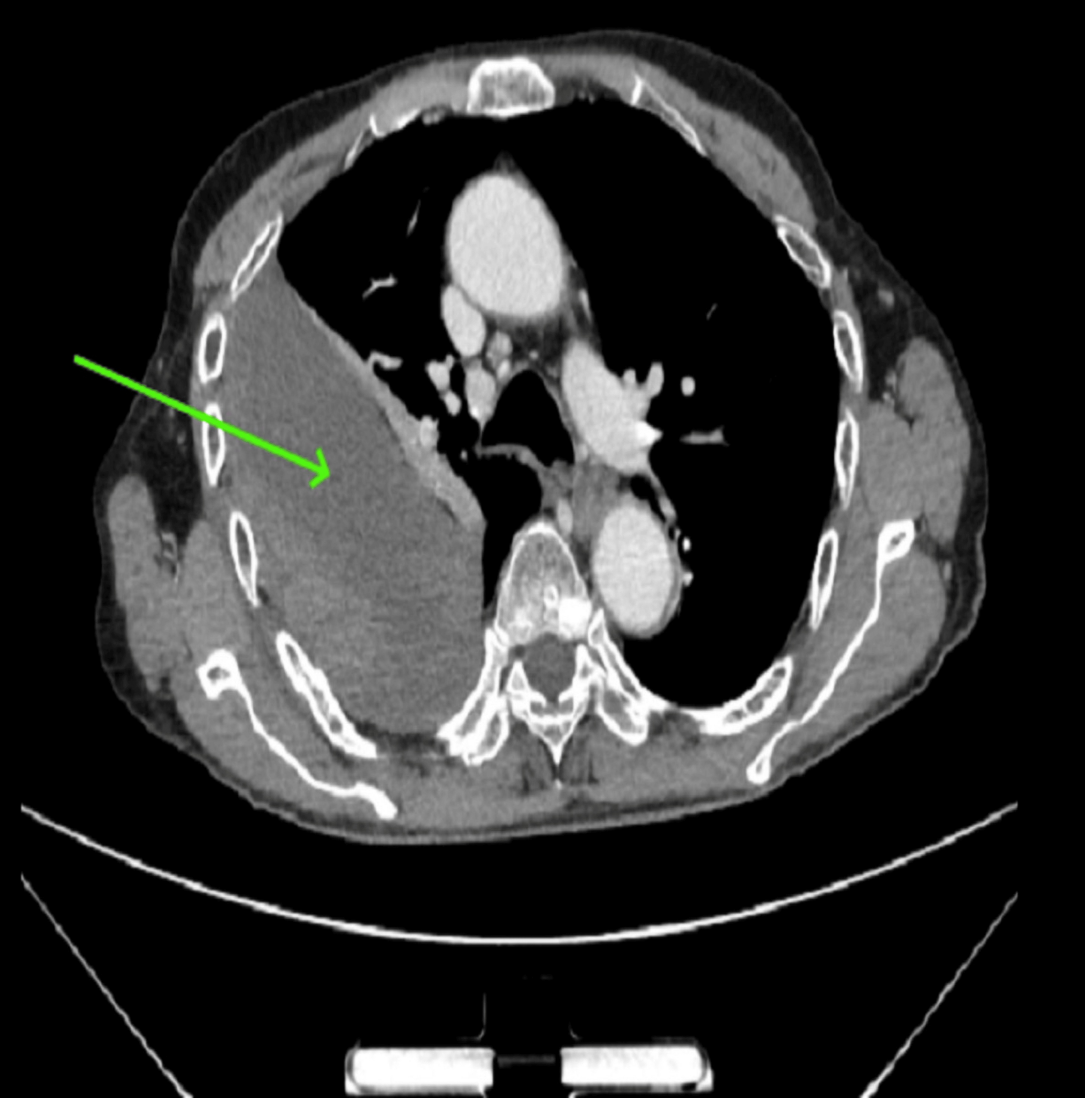
Axial section contrast-enhanced CT of the chest showing a well-defined heterodense with hyperdense extrapleural collection on the right side (green arrow) CT: computed tomography

He was then transferred for a contrast-enhanced CT scan of the thorax, which revealed a right extrapleural hematoma along with fractures of the right fifth through tenth ribs (Figure 2).

There was no evidence of a flail chest. Following these findings, the patient underwent a right-sided video-assisted thoracoscopy to evacuate the hematoma. Post-surgery, he was moved to the intensive care unit for close monitoring and further care. He showed significant improvement and was discharged on the seventh day in stable and good condition. A written consent from the next of kin was obtained.

## Discussion

Chest trauma is the third most common cause of mortality in polytrauma patients, with the first two being head trauma and abdominal trauma [4]. Bleeding between the parietal pleura and endothoracic fascia will result in extrapleural hematoma, which is usually due to bleeding intercostal vessels, or in fewer cases, bleeding internal thoracic vessels, vertebral vessels, or a ruptured thoracic aorta. A breach in the parietal pleura will result in a hemothorax [2].

The differentiation between a hemothorax and extrapleural hematoma on clinical grounds is difficult. The typical feature on a chest X-ray is the presence of a peripheral D-shaped opacity along the chest wall with the loop facing into the thorax when the extrapleural hematoma is located laterally. When it is located anteriorly or posteriorly, the chest X-ray will only show a nonspecific opacification. This opacity is not gravity-dependent, unlike a hemothorax. Blunting of the costophrenic angle is seen only if the extrapleural hematoma involves the base of the chest wall. Contrast-enhanced CT of the chest is the gold standard, which reveals a biconvex or nonconvex focal extrapleural collection with an “extrapleural fat sign.” This is the internal displacement of the extrapleural fat by the intrathoracic peripheral fluid collection. Biconvex hematomas have larger volumes. An active bleeding source can be localized in the arterial phase contrast. The ability of ultrasound to distinguish between an extrapleural hematoma and hemothorax remains to be determined [2,5-7].

Transcatheter arterial embolization (TAE) and video-assisted thoracoscopic surgery (VATS) are the minimally invasive treatment options for extrapleural hematoma. TAE has been reported to be an effective treatment option for stable patients and avoids the risks associated with an extensive operation. Patients who are unstable and patients at risk for tension physiology due to rapidly expanding hematomas are not ideal candidates for TAE. This is because TAE does not evacuate the hematoma, and thus the pressure on the mediastinal structures is not relieved [6,8,9].

The operative technique described for VATS involves intrapleural drainage of hematoma with partial pleurectomy after entering the pleural cavity [9,10]. However, in a recently described case report, the patient’s hematoma was initially drained directly without entering the thoracic cavity. This approach stabilized the patient as there was an immediate decompression of the hematoma. This was followed by exploring the pleural space for hemothorax and intrapleural drainage of the remaining hematoma with a partial pleurectomy [3]. VATS also permits the use of coagulation systems for bleeding control [11].

There are various problems encountered with the proper placement and drainage of extrapleural hematoma using an intercostal catheter. Thus, it should preferably be placed under imaging guidance. An intercostal catheter allows the administration of fibrinolytic agents for proper drainage of the hematoma [6,12].

Our patient was an elderly male who was not on any anti-platelet or anti-coagulant medications and had a large, biconvex, extrapleural hematoma originating from the lateral wall. There was no active bleeding. He sustained blunt trivial trauma to his chest around a week before his presentation to this hospital. Due to the inconsequential nature of the trauma and the mild pain, which was being controlled with oral analgesics, he did not seek any medical help. The radiologic diagnosis of the extrapleural hematoma is usually made within 24-48 hours of the initial injury, and it is quite rare for a patient to present to the hospital this late, especially if the patient had an arterial source of bleeding, as was the case in our patient [1].

## Conclusions

It is rare for an extrapleural hematoma to present with a delayed onset following thoracic trauma, as was the case with our patient. Our patient experienced only mild symptoms following minor chest trauma, which led him to avoid seeking medical care initially. It was only when he developed dyspnea that he sought medical attention, at which point he was diagnosed with an extrapleural hematoma. He was successfully managed with video-assisted thoracoscopy and evacuation of the hematoma and was discharged on day 7.

Thus, emergency physicians must be aware of this rare complication of thoracic trauma. Geriatric patients should be followed and monitored closely for the development of delayed symptoms following trivial chest traumas. There should be a low threshold to obtain repeat imaging in the event of any symptom development or worsening.
